# Relationship between SPP1 (Osteopontin) and extracellular matrix dynamics: a comprehensive review

**DOI:** 10.3389/fmed.2025.1700652

**Published:** 2025-11-10

**Authors:** Ying Liu, Xiaoyan Yan, Rui Fan

**Affiliations:** 1 Department of Pulmonary Medicine, Xiangyang Hospital Affiliated with Hubei University of Traditional Chinese Medicine, Xiangyang, Hubei, China; 2 Department of Geriatric Medicine, Affiliated Hospital of Shandong University of Traditional Chinese Medicine, Jinan, Shandong, China; 3 Department of Respiratory and Critical Care Medicine, Provincial Hospital Affiliated to Shandong First Medical University, Jinan, Shandong, China

**Keywords:** SPP1, Osteopontin, extracellular matrix (ECM), SPP1^+^ macrophages, epithelial-mesenchymal transition (EMT)

## Abstract

**Background:**

Secreted Phosphoprotein 1 (SPP1), which encodes Osteopontin, a key member of the SIBLING family, is a multifunctional ECM glycoprotein and cytokine. Its interaction with collagen and other ECM components drives pathological remodeling across multiple diseases, yet a unified mechanistic framework remains elusive.

**Objective:**

This review synthesizes current evidence on SPP1-mediated ECM dysregulation, focusing on collagen deposition, epithelial-mesenchymal transition (EMT), and fibrosis, with the goal of elucidating its role as a central pathological hub.

**Methods:**

We synthesize the findings from multi-omics analyses (single-cell RNA sequencing, spatial transcriptomics), machine learning, and *in vivo*/*in vitro* experimental studies, aiming to elucidate the role of SPP1 (Osteopontin) in the dysregulation of the extracellular matrix (ECM) across various diseases via a systematic literature review (1990–2025).

**Conclusion:**

SPP1 is a master regulator of pathological ECM dynamics, driven by conserved SPP1^+^ macrophage-ECM interactions. Targeting the SPP1-collagen axis may offer unified strategies for fibrosis and metastasis suppression. Future work should prioritize *in vivo* validation in osteoarthritis and clinical translation of SPP1-directed therapies.

## 1 Introduction

Secreted Phosphoprotein 1 (SPP1), which encodes Osteopontin, is a member of the small integrin-binding ligand N-linked glycoprotein (SIBLING) family of secreted phosphoproteins that participate in bone mineralization (1). The SIBLING family consists of five secreted phosphoglycoproteins: secreted phosphoprotein 1 (SPP1), bone sialoprotein (BSP), dentin matrix protein-1 (DMP1), dentin sialophosphoprotein (DSPP), and matrix extracellular phosphoglycoprotein (MEPE) (2).

SPP1 was first cloned and sequenced by Kiefer et al. (3), revealing a conserved Arg-Gly-Asp (RGD) cell adhesion motif (4). Its mRNA is mainly expressed in osteocytes and the decidua during early stages pregnancy. Kohri et al. (5, 6) demonstrated that Osteopontin, which constitutes the urinary stone matrix, is upregulated in the renal distal tubules and actively participates in calcium oxalate stone formation. Subsequent research confirmed SPP1 expression in normal adult human and monkey kidneys, specifically localized to the distal convoluted and straight tubules in both the cortex and medulla (7). The authors further demonstrated the colocalization of SPP1 with MMPs in human eccrine sweat gland cells, mostly perinuclearly, a pattern consistent with SIBLING family members and their metalloproteinase counterparts; neither SPP1 nor MMPs was identified in the sweat gland stroma or monkey lacrimal gland structures. Shinohara et al. (8) discovered two translational initiation sites in mouse SPP1 mRNA, producing a full-length isoform (including a signal peptide) and a truncated isoform (devoid of signal peptide). These correspond to 75 kDa and 70 kDa proteins in dendritic cells, exhibiting different subcellular localizations: the full-length isoform is transported to secretory vesicles and the Golgi apparatus, while the short isoform is predominantly localizes in the cytoplasm (8).

The Osteopontin encoded by SPP1 promotes the adherence of osteoclasts to the calcified bone matrix, which is intricately associated with the dynamics of the extracellular matrix (ECM) (9). Osteopontin exhibits a strong affinity for the binding with hydroxyapatite. Additionally, the osteoclast vitronectin receptor is situated in the cell membrane. It may engage in binding with the SPP1 protein, which acts as a cytokine that stimulates the synthesis of IFN-*γ* and IL-12, which promotes EMT and ECM deposition. In particular, SPP1-positive macrophages (SPP1^+^ macrophages) refer to a subset of macrophages characterized by increased expression of SPP1, identified through immunohistochemistry, flow cytometry, or single-cell RNA sequencing (10). SPP1^+^ macrophages are common in pathological microenvironments, including fibrosis and malignancies, and play a significant role in matrix remodeling (11). These macrophages produce large amounts of Osteopontin, which acts as matrix-associated glycoprotein, involved in the dynamic regulation of the ECM by facilitating fibrotic deposition, and managing cell-matrix adhesion. In recent years, SPP1 and SPP1^+^ macrophages have garnered increasing attention in various disease settings. SPP1^+^ macrophages not only play a crucial role as tumor-associated macrophages (TAMs) within the tumor microenvironment (12–15) but also participate in physiological processes, such as aging (16), and in a wide range of non-cancerous diseases, including rheumatoid arthritis (17), neurodegeneration (18, 19), and fibrosis (20, 21). This perspective provides a theoretical foundation for a deeper understanding of the mechanisms by which SPP1 regulates ECM dynamics and its role in disease (22).

**Figure 1:**
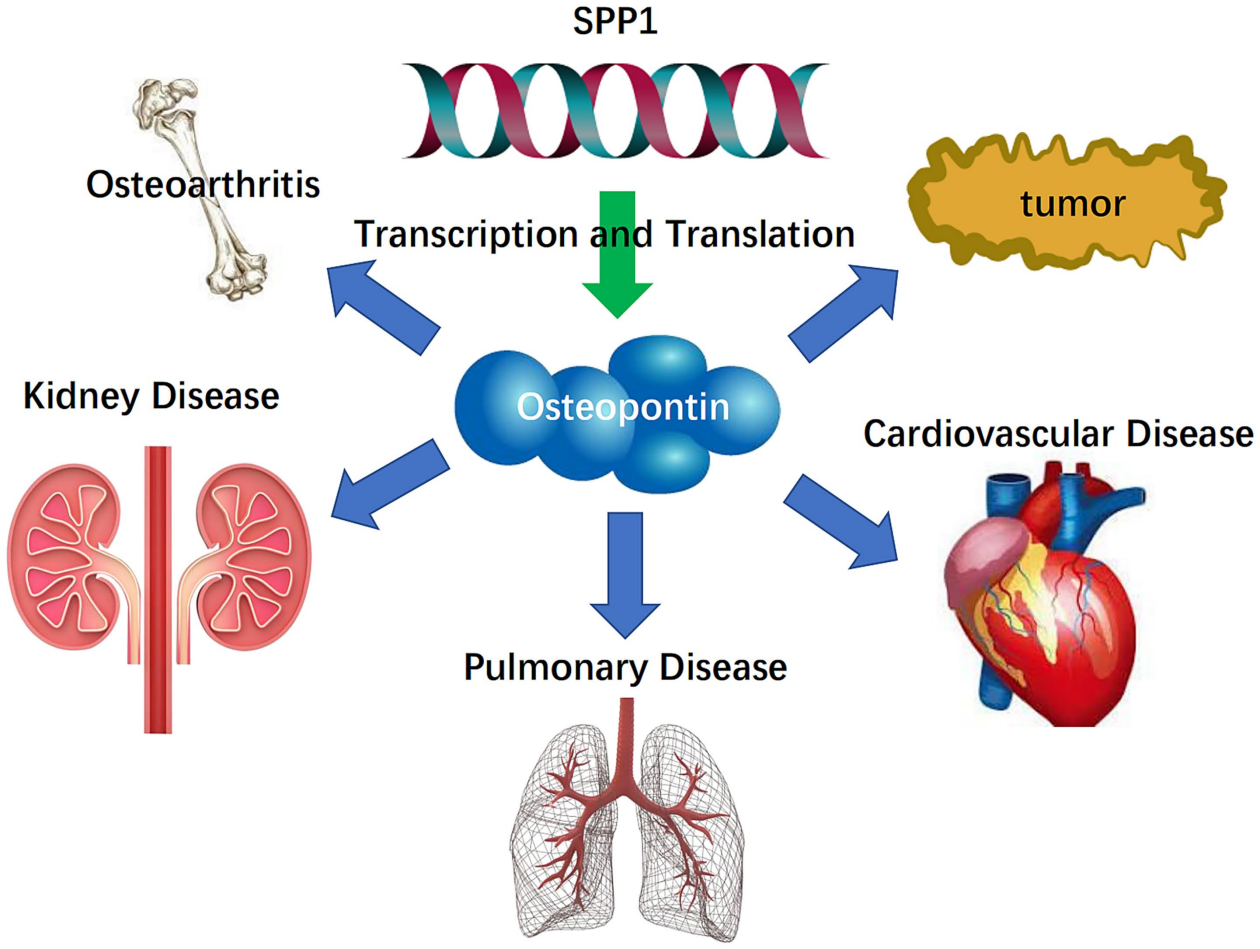
Flowchart of the systematic review process on the relationship between SPP1 (Osteopontin) and ECM.

SPP1 and the Osteopontin it encodes are crucial factors influencing ECM dynamics and play a significant role in tumor diseases, cardiovascular diseases, pulmonary diseases, chronic kidney disease, and osteoarthritis (23). It will be introduced in further detail below. The research process is shown in Figure 1.

## 2 Molecular architecture, function, and signaling of SPP1 (Osteopontin)

The SIBLING protein family is a class of evolutionarily conserved, structurally related ECM glycoproteins. SPP1 is one of the main members of this protein family and is involved in the deposition of the ECM and EMT mechanisms. Each SIBLING protein has a minimum of one “acidic, serine- and aspartic acid-rich motif” (ASARM) and many Ser-x-Glu/pSer sequences that, upon phosphorylation, facilitate biomineralization and ECM deposition. Osteopontin functions by regulating the nucleation and development of hydroxyapatite crystals. The biological activities of Osteopontin are facilitated through interactions with cell surface integrins and the activation of certain MMPs, therefore coordinating a complicated relationship between cellular signaling and ECM remodeling in mineralized tissues (24).

**Figure 2:**
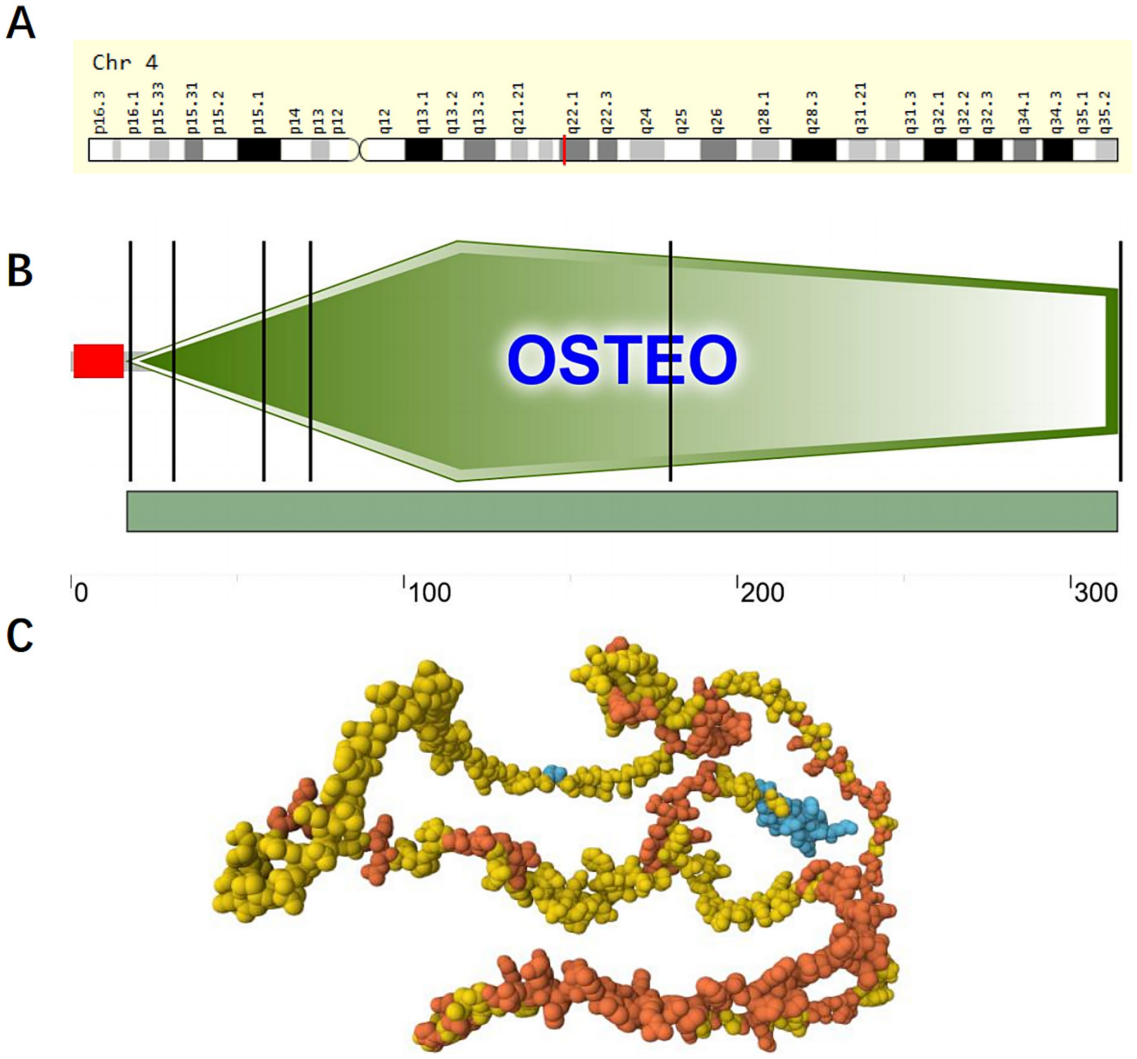
Domains within *Homo sapiens* protein SPP1 (OSTP_HUMAN, P10451). **(A)** Location of SPP1 on chromosome 4; **(B)** Schematic representation of SPP1; The red portion represents the signal peptide. The green portion represents the OSTEO domain. **(C)** AlphaFold Model of SPP1 (AF-P10451-F1), model confidence: dark blue: very high (pLDDT >90); light blue: confident (90 > pLDDT >70); yellow: low (70 > pLDDT >50); orange: very low (pLDDT <50), AlphaFold produces a per-residue confidence score (pLDDT) between 0 and 100. Some regions below 50 pLDDT may be unstructured in isolation.

The SPP1 is located in the 4q21-q25 region of chromosome 4, with genomic coordinates (GRCh38): 4:87,975,714–87,983,411 (25), as shown in Figure 2A. According to the Genecard database, SPP1 is a protein-coding gene. SPP1 is associated with various cancers, cardiovascular diseases, respiratory diseases, and kidney diseases. Related pathways include the integrin pathway and ERK signaling pathway. Gene Ontology (GO) annotations associated with this gene cover cytokine activity and ECM binding (26). SPP1 demonstrates significant evolutionary conservation among several species. The SPP1 which encoding Osteopontin consists of seven exons featuring canonical splice sites. While genetic linkage studies initially linked the SPP1 locus to dentinogenesis imperfecta type II, further investigation ruled out coding sequence mutations within its exons as the direct cause of the disease (27). Research shows that SPP1 serves as a specific marker for a profibrotic macrophage subpopulation that expands dramatically in idiopathic pulmonary fibrosis (IPF). SPP1^+^ macrophages, characterized by co-expression of MERTK, promote fibrosis through Osteopontin deposition and aberrant repair processes, positioning SPP1 as a central mediator and potential therapeutic target in IPF (28).

Osteopontin, a multifunctional ECM protein, serves dual roles in bone remodeling by facilitating cell-matrix adhesion and in preventing pathological calcification. Baccarani believe that Osteopontin functions as a potent inhibitor of pathological calcification by localizing within elastic fibers of the aorta and skin, where it helps prevent mineral precipitation (29). Osteopontin expression is strongly and specifically induced by elevated extracellular phosphate levels, a product of alkaline phosphatase activity. Beck (30) found that Osteopontin expression is strongly and specifically induced by elevated extracellular phosphate levels, a product of alkaline phosphatase activity, elucidating the relationship between Osteopontin and phosphate. The molecular architecture of Osteopontin remains incompletely elucidated; however, its fundamental composition includes a signal peptide and an OSTEO domain., as shown in Figures 2B,C and Tables 1, 2.

**Table 1:** Confidently predicted domains, repeats, motifs and features.

Name	Start	End	E-value
Signal peptide	1	16	N/A
OSTEO-domain	17	314	1.21e-187

**Table 2:** Features NOT shown in the diagram.

Name	Start	End	E-value	Reason
Pfam: Osteopontin	21	314	2.6e-161	Overlap
Low complexity	81	131	N/A	Overlap
Low complexity	272	282	N/A	Overlap

**Figure 3:**
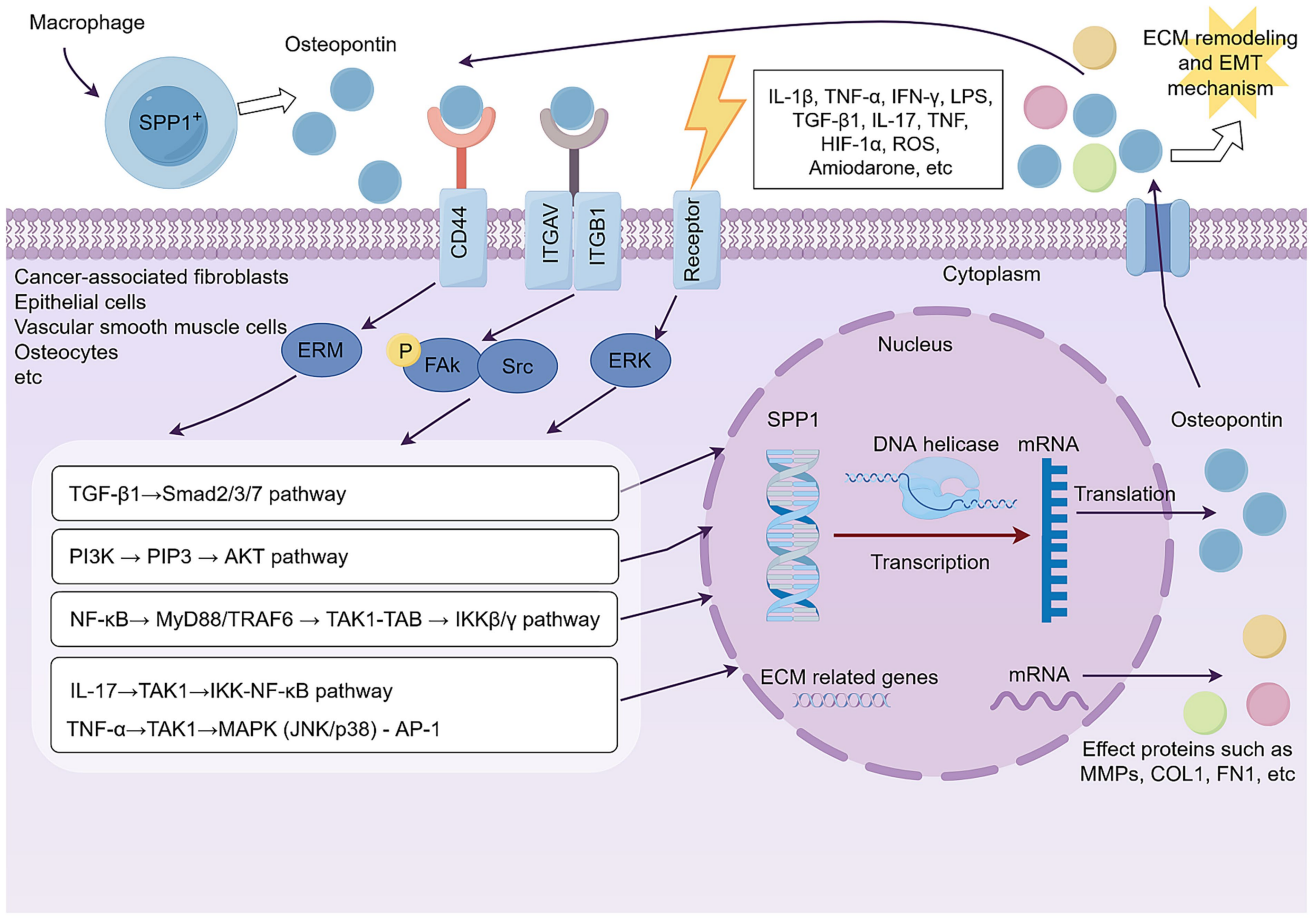
SPP1 (Osteopontin) secreted by SPP1^+^ macrophages orchestrate a downstream signaling network that converges to drive pathological ECM remodeling and EMT mechanism in target cells. By Figdraw.

SPP1 (Osteopontin), an essential stromal cell factor, facilitates EMT mechanism and ECM remodeling via many molecular signaling pathways. Osteopontin is predominantly secreted by a distinct SPP1^+^ macrophage subset located inside the disease microenvironment, and its mode of action commences post-secretion. Secreted Osteopontin then undergoes ligand-receptor interactions with integrins (such as ITGAV/ITGB1) and CD44 on the surfaces of target cells, such as fibroblasts, epithelial cells, and vascular smooth muscle cells. This binding triggers signaling by the ERM protein family or phosphorylated FAK-Src signaling. Downstream signaling converges on pro-fibrotic and pro-inflammatory pathways, including the TGF-*β*1/Smads, PI3K/AKT, and NF-κB axes. The TGF-*β*1/Smads pathway is a major driver of the fibrotic response and influences ECM dynamics, while the PI3K/AKT pathway promotes cellular activity, metabolic reprogramming, and the synthesis of specific proteins. These downstream pathways are extensively interconnected, forming a highly integrated and collaborative network. For example, the synergistic relationship between NF-κB and the TGF-*β*1/Smads pathways exists. Proinflammatory NF-κB signaling can enhance the TGF-*β*1-driven fibrotic response, while TGF-*β*1 itself can activate the NF-κB pathway in a non-canonical manner. The integration of these signals ultimately leads to the nuclear translocation of key transcription factors (such as NF-κB, the Smads complex, and *β*-catenin), which synergistically bind to the promoter regions of SPP1 and ECM-related genes. This transcriptional reprogramming induces physiological changes such as EMT mechanism, ECM remodeling, and myofibroblast activation, establishing a pathological positive feedback loop. The signaling mechanism of SPP1 (Osteopontin) is shown in Figure 3.

## 3 Relationship between SPP1 (Osteopontin) and ECM dynamics in tumor growth and metastasis

According to the 2025 Cancer Statistics report published by the American Cancer Society, data from the US national cancer registry and demographic analyses indicate a continuing rise in the overall cancer burden, with an estimated 2,041,910 new cancer cases projected in 2025 (31). Notably, the following cancer types exhibit significantly high incidence rates: lung cancer, colorectal cancer, pancreatic ductal adenocarcinoma, gastric cancer, breast cancer, head and neck squamous cell carcinoma, prostate cancer, and hepatocellular carcinoma. Extensive independent studies have demonstrated that SPP1, as an oncogene, promotes the progression of these malignancies, particularly through its role in mediating pathological remodeling of the ECM across these cancer types. Table 3 illustrates the function of SPP1 in tumor growth and metastasis.

**Table 3:** The function of SPP1 in tumor growth and metastasis.

Gene	Disease	Expression	Study type	Sample	References
SPP1	Lung cancer (NSCLC)	↑	In vivo	Human patients	(32)
	Colon cancer	↑	In vivo and in vitro	Human patients and Human cell (HT-29)	(33)
	Colorectal cancer	↑	In vivo studies integrating bioinformatics	Human patients	(34)
	Pancreatic ductal Adenocarcinoma (PDAC)	↑	Bioinformatics-led in vivo studies	Human patients	(13)
	Gastric cancer (GC)	↑	Bioinformatics-led in vivo studies	Human patients	(35)
	Triple-negative breast cancer (TNBC)	↑	In vivo, in vitro, and bioinformatics	Human patients	(36)
	Head and neck squamous cell carcinoma (HNSCC)	↑	Bioinformatics and clinical validation	Human patients	(37)
	Prostate cancer	↑	*In vivo*, in vitro, and bioinformatics	Human patients and Human cell (Organoids)	(38)
				Human patients and murine models	(39)
				Human patients, animal cell, and animal models	(40)
				Human patients	(41)
	Hepatocellular carcinoma (HCC)	↑	Bioinformatics	Human patient genomic and clinical data	(42)

Rouanne et al. (32) demonstrated through clinical cohort analysis that elevated serum levels of SPP1, an ECM protein, significantly correlate with tumor progression in non-small cell lung cancer (NSCLC). Their data revealed that each 50 ng/mL increase in serum SPP1 was associated with a 69% higher risk of metastasis (HR 1.69, 95% CI 1.12–2.56, *p* = 0.01) and 95% increased mortality risk (HR 1.95, 95% CI 1.15–3.32, *p* = 0.01), suggesting SPP1-mediated ECM dysregulation may drive malignant progression. Gómez de Segura et al. (33) demonstrated through integrated clinical and *in vitro* analyses that decreased expression of microfibril-associated glycoprotein-1 (MAGP-1, encoded by MFAP2 gene) in obesity-associated colon cancer leads to dysregulated TGF-*β*1 signaling and subsequent upregulation of SPP1. Their work revealed that SPP1-mediated ECM remodeling promotes collagen VI (COL6A3) and decorin (DCN) deposition, creating a fibrotic tumor microenvironment that physically excludes cytotoxic T lymphocytes (CTLs) and contributes to immune evasion. This study provides mechanistic insight into how metabolic dysregulation in obesity drives tumor progression through the SPP1-ECM axis (33). Through scRNA-seq analysis of liver tissues from Microsatellite stable metastatic-type metastatic colorectal cancer (MSS-mCRC), normal liver tissues, and PBMCs, Sathe et al. (34) revealed that SPP1^+^ macrophages in the metastatic tumor microenvironment (TME) secrete SPP1, which binds to integrin receptors (ITGAV/ITGB1) on cancer-associated fibroblasts (CAFs). This interaction activates CAFs to produce excessive collagen, ECM glycoproteins (e.g., FN1), and remodeling enzymes (e.g., MMPs, LOXL2), driving aberrant ECM deposition. The resulting dense ECM promotes MSS-mCRC progression and therapy resistance by impairing CD8^+^ T cell infiltration, increasing matrix stiffness to facilitate invasive metastasis, and inducing angiogenesis (34). Evan et al. demonstrated that SPP1 primarily promotes ECM deposition and EMT by orchestrating the crosstalk between TAMs and myofibroblastic cancer-associated fibroblasts (myCAFs). This interaction fosters an immunosuppressive tumor microenvironment (TME), ultimately driving the progression of pancreatic ductal adenocarcinoma (PDAC) (13). Thus, SPP1 emerges as a central regulator of tumor malignancy by orchestrating pathogenic ECM remodeling. Su et al. uncovered the heterogeneity of the tumor immune microenvironment (TiME) in gastric cancer (GC) patients with different mismatch repair (MMR) statuses. Their study highlights that the proficient MMR (pMMR) TiME is characterized by hypoxia, pro-angiogenic signaling, and ECM remodeling, driven by the presence of GC2 cells, SPP1^+^ macrophages, FAP + fibroblasts, and E2 endothelial cells. These findings are critical for developing targeted immunotherapies tailored to pMMR GC patients (35). By integrating single-cell transcriptomics, spatial multi-omics, and functional validation, Lu et al. elucidated a hypoxia-mediated immunosuppressive axis in triple-negative breast cancer (TNBC), wherein SPP1^+^ macrophages orchestrate tumor progression through dual secretion of SPP1 and TGF-*β*1. These cytokines directly program stromal fibroblasts to differentiate into ECM-producing CAFs (ecmCAFs), which in turn drive pathological ECM remodeling featuring excessive collagen deposition and stromal fibrosis (36). Through integrative bioinformatics analysis of multiple datasets (GSE6791, GSE29330, GSE58911), Cheon et al. identified SPP1 as a hub gene significantly upregulated in head and neck squamous cell carcinoma (HNSCC). Their study demonstrated that SPP1 expression is directly associated with functional enrichment of ECM organization and degradation processes. Clinically, elevated SPP1 expression correlated with advanced tumor grade, progressive clinical stage, and poor prognosis in HNSCC patients (37). Pang et al. established SPP1 as a pivotal molecular nexus linking ECM remodeling to metastatic castration-resistant progression in prostate cancer (mCRPC). Their integrative multi-omics and organoid approach revealed that SPP1 orchestrates a feedforward loop: it not only activates Androgen receptor (AR) signaling to promote therapy resistance but also directly mediates ECM reorganization through collagen crosslinking and fibronectin assembly. This dual functionality creates a permissive microenvironment for metastatic dissemination, positioning SPP1-ECM crosstalk as a promising therapeutic target in mCRPC (38). Similarly, the role of SPP1 in prostate cancer has been confirmed in several other studies (39–41). Based on molecular subtyping of the ECM in the TCGA-LIHC cohort, SPP1 was identified as a core gene for constructing an ECM-related prognostic model in hepatocellular carcinoma (HCC). High SPP1 expression marked pathological ECM remodeling features and was significantly associated with advanced histological grade, resistance to immunotherapy, and poor prognosis. These findings provide a mechanistic rationale for targeting the SPP1-ECM axis to reprogram the immunosuppressive tumor microenvironment in HCC (42). In summary, SPP1 acts as a pivotal oncogenic driver by orchestrating pathological remodeling of the ECM during tumorigenesis and progression across multiple malignancies. Cross-cancer analyses demonstrate that SPP1-mediated collagen deposition, stromal fibrosis, and integrin-dependent ECM receptor signaling collectively fuel metastatic dissemination and therapy resistance.

## 4 Relationship between SPP1 (Osteopontin) and ECM dynamics in cardiovascular disease

Cardiovascular disease (CVD) is the leading cause of death worldwide, accounting for approximately 18.5 million deaths (9.6 million men and 8.9 million women), or about one-third of all deaths globally (43). CVD is also the leading cause of death in China, accounting for nearly 4 million deaths, highlighting its enormous disease burden and risk of premature death, which is on the rise year by year (44). Numerous studies suggest that SPP1 may contribute to the development and progression of cardiovascular diseases, including atherosclerosis, abdominal aortic aneurysm, dilated cardiomyopathy with heart failure, thoracic aortic dissection, and myocardial infarction, via the EMT pathway. Table 4 illustrates the function of SPP1 in Cardiovascular Disease.

**Table 4:** The function of SPP1 in cardiovascular disease.

Gene	Disease	Expression	Study type	Sample	References
SPP1	Atherosclerosis (AS)	↑	In vivo mechanism studies based on multi-omics integration	Human patients	(45)
				Human patient genomic data	(46)
	Abdominal aortic aneurysm (AAA)	↑	In vivo and in vitro	Human patients and murine models	(47)
				Murine models, animal cells, and primary human cells	(48)
	Dilated cardiomyopathy with heart failure (DCM-HF)	↑	In vivo (Clinical sample-driven multi-omics research)	Human patients	(49)
	Aortic dissection (TAD)	↑	In vivo and in vitro	Murine models and primary murine cells	(50)
	Myocardial infarction (MI)	↑	Bioinformatics	Human patient genomics data	(51)

Single-cell and spatial analyses revealed a significant interaction between SPP1^+^ macrophages and ITLN1^+^ foam cells, mediated by the SPP1-CD44 ligand-receptor axes, which accelerates arterial lipid accumulation and EMT transformation, which is necessary to develop effective immunotherapeutic strategies against atherosclerosis (AS) (45). LASSO regression and SVM-REF also verified the pivotal role of SPP1 in atherosclerosis (46). Markus et al. found that platelets play a key role in promoting the formation of abdominal aortic aneurysm (AAA) by regulating inflammation and degrading the ECM. Platelets are responsible for upregulating the expression of the SPP1 gene in macrophages and aortic tissue, which triggers inflammation and remodeling, while promoting platelet adhesion and migration to the abdominal aortic wall and intraluminal thrombus (ILT) (47). It was found that SPP1 is upregulated in vascular smooth muscle cells (VSMCs) induced by Di-(2-ethylhexyl) phthalate (DEHP), and its phenotypic switch is significantly accelerated, indicating that M1 macrophage polarization and VSMC phenotypic switch can exacerbate the progression of AAA, which also involves the EMT process (48). Furthermore, A study analyzed the expression levels of cardiovascular-related proteins in patients with dilated cardiomyopathy with heart failure (DCM-HF) (*n* = 20) and healthy controls (Normal) (*n* = 18). Using Olink proteomics analysis, five key proteins, including SPP1, were identified and validated in human serum samples via ELISA, indicating that SPP1 is equally important in DCM-HF and may be involved in the EMT mechanism of the disease (49). Similarly, Suwei et al. established a thoracic aortic dissection (TAD) mouse model by perfusing angiotensin (Ang) II into mice administered *β*-aminopropionitrile. Through mouse experiments, they confirmed that upregulation of SPP1 is a key sign of the pathological phenotype transition of aortic smooth muscle cells from a contractile state to a synthetic state, and IGFBP3 can directly inhibit this process, thereby maintaining vascular homeostasis (50). Finally, GEO database analysis showed that SPP1, as a biomarker for myocardial infarction (MI), participated in fibroblast proliferation and myocardial remodeling, and was also involved in the EMT mechanism (51). In summary, SPP1 coordinates immune cell interactions, drives cell phenotypic transformation, and promotes pathological tissue remodeling, thereby affecting ECM dynamics and becoming a common pathogenic factor in the progression of multiple cardiovascular diseases.

## 5 Relationship between SPP1 (Osteopontin) and ECM dynamics in pulmonary disease

Data from the Worldwide Burden of Disease Study indicate that chronic respiratory disorders (CRDs) pose a significant global health challenge. Annually, they result in the deaths of 4.4 million individuals and impact approximately 468 million people. From 1990 to 2021, the age-standardized prevalence rate decreased by 1.01%, whereas it increased by 0.20% during the pandemic from 2019 to 2021, exhibiting significant regional disparities in risk factors (52). Numerous studies indicate that SPP1-mediated ECM remodeling and EMT mechanisms are the primary determinants in the onset of several chronic lung diseases, including pulmonary fibrosis, silicosis, sarcoidosis, and chronic airway diseases. Table 5 illustrates the function of SPP1 in Pulmonary Disease.

**Table 5:** The function of SPP1 in pulmonary disease.

Gene	Disease	Expression	Study type	Sample	References
SPP1	Idiopathic pulmonary fibrosis (IPF)	↑	In vivo, in vitro, and bioinformatics	Murine models and murine cells	(53)
				Human patients, in vitro cell models, and animal model	(54)
	Multi-organ fibrosis	↑	Bioinformatics research based on multi-omics integration	Human patients and murine models	(55)
	COVID-19-associated Rapid pulmonary fibrosis	↑	In vivo, in vitro, and bioinformatics	Human patients, murine models, and in vitro cell models	(56)
	Silica-induced lung injury.	↑	Bioinformatics research on spatial omics integration	Murine models and murine cells	(57)
	Sarcoidosis	↑	In vivo, in vitro, and bioinformatics	Human patients and human cells	(58)
				Human patients and in vitro cell models	(59)
				Human patients	(60)
	Chronic obstructive pulmonary disease (COPD)	↑	In vivo and in vitro	Human patients and murine models	(61)
				Human patients	(62)
	Asthma	↑	In vivo and bioinformatics	Human patients	(63)
				Human patients and murine models	(64)
				Human patients	(65)
		↓	In vivo and bioinformatics	Human patients and murine models	(66)
				Murine models and murine cells	(67)

Single-cell analysis verified that monocyte-derived interstitial macrophages (Mo-IMs) exhibit a pro-fibrotic phenotype in the initial stages of pulmonary fibrosis and engage with fibroblasts via the SPP1 signaling pathway, facilitating EMT and ECM deposition, thereby advancing disease progression (53). Conversely, knockdown of SPP1 expression inhibits macrophage-induced EMT in epithelial cells and fibroblasts. *In vivo* treatment with an SPP1 inhibitor enhances lung function and ameliorates idiopathic pulmonary fibrosis (IPF). Inhibiting SPP1 expression *in vivo* effectively mitigates the progression of IPF, suggesting that SPP1 in macrophages may be a potential therapeutic target for IPF (54). Furthermore, SPP1^+^ macrophages demonstrate a conserved matrisome-associated macrophage (MAM) polarization in multi-organ fibrosis, particularly in pulmonary and liver fibrosis, directly facilitating fibrosis via ECM remodeling and metabolic reprogramming, thereby uncovering novel targets for cross-tissue intervention (55). Integrated multi-omics analyses of COVID-19-associated Rapid pulmonary fibrosis (RPF) patients and murine models reveal that CD163^+^ macrophages drive rapid pulmonary fibrosis progression via SPP1 secretion, identifying the CD163^+^-SPP1 axis as a potential therapeutic target (56). Tao et al. (57) identified a unique stress-induced epithelial subset (C0) that enhances immunological crosstalk, activates EMT pathways, increases ECM deposition, and facilitates tissue remodeling in silica-induced lung damage via the SPP1-CD44 signaling pathway. Therefore, SPP1, as a common molecular hub connecting immune cells, epithelial cells and ECM remodeling, plays a core role in the process of pulmonary fibrosis driven by different causes. SPP1 is raised in the blood and granulomatous tissue of individuals with sarcoidosis, elucidating the EMT pathway in disease development (58, 59). Research indicates that single-nucleotide polymorphisms (SNPs) in the SPP1 gene are strongly associated with susceptibility to pulmonary lesions in sarcoidosis, which also encompasses the EMT pathway (60). In chronic obstructive pulmonary disease (COPD), SPP1 expression is markedly increased, particularly in airway cells and antigen-presenting cells of patients with emphysema. This elevation facilitates Th1/Th17-mediated inflammation and Th2-mediated inflammatory responses, promotes neutrophil recruitment, and leads to MMP9-dependent tissue destruction (61, 62). SPP1 expression is increased in serum, sputum, and bronchial tissue in asthma, correlating with disease severity, late-onset asthma, and airway remodeling (63). It can promote Th2 inflammation, smooth muscle proliferation, and collagen deposition through pathways such as PI3K/AKT, thus exacerbating the disease (64, 65), while also exhibiting anti-inflammatory properties at specific stages. In a house dust mite (HDM) induced allergic asthma mouse model, SPP1/Osteopontin was shown to significantly enhance the host’s ability to defend against *Streptococcus pneumoniae* infection by inhibiting airway inflammatory cell infiltration, alleviating tissue damage, and reducing proinflammatory cytokine levels, as manifested by a significant reduction in bacterial load in alveolar lavage fluid and lung tissue (66). Furthermore, Samitas (67) found that SPP1/Osteopontin plays a critical role in the modulation of allergic asthma by preserving the homeostasis of the gut-lung axis. Its deficiency leads to increased airway inflammation, which is associated with gut barrier dysfunction, microbiota dysbiosis, and the PD-1/PD-L1-mediated disruption of the Treg/Th17 balance, as evidenced by fecal microbiota transplantation and pathway analysis. Therefore, SPP1 plays a dual role in enabling ECM remodeling and EMT processes in asthma and COPD. In summary, SPP1 constitutes a common core pathogenic mechanism of various chronic lung diseases by driving ECM remodeling, EMT and immune inflammation regulation.

## 6 Relationship between SPP1 (Osteopontin) and ECM dynamics in kidney disease

The Lancet Global Burden of Disease Study indicates that chronic kidney disease (CKD) impacts over 700 million individuals globally (prevalence rate 9.1%), ranks as the 12th greatest cause of mortality (constituting 4.6% of global deaths), and represents a significant threat to human health (68). SPP1 affects the occurrence, development, and prognosis of CKD by affecting ECM dynamics, and is also important in the process of acute kidney injury to chronic kidney disease (AKI-CKD) transformation. Table 6 illustrates the function of SPP1 in Kidney Disease. Table 6 illustrates the function of SPP1 in Kidney Disease.

**Table 6:** The function of SPP1 in kidney disease.

Gene	Disease	Expression	Study type	Sample	References
SPP1	Chronic kidney disease (CKD)	↑	In vivo, in vitro, and bioinformatics	Human patients, murine models, and animal cells	(69)
				Murine models and murine cell line (C2C12)	(70)
				Human patients	(71)
	Acute kidney injury to chronic kidney disease (AKI-CKD)	↑	In vivo and bioinformatics	Murine models and murine cells	(72)
				Murine models and murine cells	(73)

CKD is defined as a syndrome marked by a sustained reduction in glomerular filtration rate lasting at least three months, accompanied by tubular atrophy, interstitial fibrosis, and advancing systemic complications. The pathological progression is influenced by epigenetic regulation and intercellular vesicle signaling. Research indicates that circulating small extracellular vesicles (sEVs) originating from CKD promote pathological calcification of vascular smooth muscle cells (VSMCs) by depleting protective miRNAs, which in turn releases the inhibition of VEGFA signaling, marked by a significant upregulation of genes such as SPP1 (69). An additional investigation revealed the significant function of SPP1 in modulating ECM dynamics in chronic kidney disease, accompanied by sarcopenia. In animal experiments on CKD, increased secretion of SPP1 by the kidneys, which circulates to skeletal muscle, directly activating the expression of the muscle atrophy marker Murf-1 and promoting smaller myotubes. Pharmacological inhibition of SPP1 *in vivo* significantly increases the weight of the gastrocnemius and tibialis anterior muscles, improves the atrophy phenotype, and reprograms the muscle transcriptome, thereby confirming SPP1 as the central pathogenic factor and therapeutic target in the CKD-muscle axis (70). Another study involving the European CKD population genome confirmed that genetic variation upstream of the SPP1 influences the progression of CKD by directly regulating Osteopontin expression levels. This mechanism has been validated through rare variant aggregation analysis and multi-level cross-cohort assessments (71). Furthermore, SPP1 is integral to the progression from acute kidney injury to chronic kidney disease (AKI-CKD). Research in single-cell transcriptomics has identified SPP1 as a crucial hub molecule in polyploid cells during the transition from acute kidney injury to chronic kidney disease, involving the EMT mechanism. *In vivo* gene deletion of SPP1 enhances renal fibrosis via influencing ECM dynamics, substantiating the targeting of SPP1 to impede the advancement of renal fibrosis (72). A related study confirmed that in NF-κB deficient mouse models, the expression of the key inflammatory factor SPP1 in proximal tubule cells (FR-PTCs) was significantly reduced, resulting in decreased pathological damage associated with AKI-CKD (73). This establishes a molecular foundation for the inhibition of SPP1 and other NF-κB effector molecules to impede the advancement of AKI-CKD. In summary, SPP1 establishes its core position in CKD and AKI-CKD transformation by driving ECM remodeling and affecting ECM dynamics, and is a highly potential pleiotropic therapeutic target.

## 7 Relationship between SPP1 (Osteopontin) and ECM dynamics in osteoarthritis

Osteoarthritis (OA) is a very debilitating chronic joint disorder marked by the progressive deterioration of articular cartilage, synovial inflammation, and remodeling of subchondral bone. The pathogenic process is influenced by an imbalance of mechanical stress, immunological metabolic abnormalities, and hereditary variables. The China OA Disease Burden Study (1990–2021) revealed that the age-standardized incidence rate (ASIR), prevalence rate (ASPR), and disability-adjusted life rate (ASDR) showed a continuous upward trend, and are predicted to continue to rise by 2050 (74). Table 7 illustrates the function of SPP1 in OA.

**Table 7:** The function of SPP1 in osteoarthritis.

Gene	Disease	Expression	Study type	Sample	References
SPP1	Osteoarthritis	↑	In vivo and multi-omics approaches	Human patients	(75)
				Human primary cells	(76)
				Human genomic data and rat models	(77)

He et al. discovered through multi-omics integrated analysis and machine learning that SPP1 serves as a pivotal immunological metabolic hub gene in OA progression, instigating the aberrant activation of EMT-related pathways. PCR verification indicated considerable overexpression, and the predictive model developed as a key marker may effectively distinguish OA subtypes (75). Another study utilizing a simulated microgravity model of the human meniscus discovered that mechanical unloading of the knee articular cartilage initiates the inflammation-calcification cascade by specifically upregulating SPP1 expression in chondrocytes, highlighting its sex-specific regulatory role as a central factor in the perception of mechanical stress and subsequent pathological transformation (76). Similarly, Yang et al. found that SPP1, as a core molecule of OA, drives EMT by synergizing with the IL-17/TNF inflammatory pathway, providing a new intervention framework for targeting SPP1 to regulate OA immune metabolism (77). Unfortunately, most studies on the role of SPP1 in osteoarthritis are based on bioinformatics analysis, with few *in vivo* experiments. We look forward to subsequent researchers conducting more in vivo studies to provide more evidence that SPP1 affects osteoarthritis through ECM dynamics. Therefore, SPP1, as a hub molecule connecting mechanical stress, inflammatory signals and ECM remodeling, plays a core role in the progression of osteoarthritis, but its precise mechanism requires additional validation through in vivo research.

## 8 Conclusion

SPP1 (Osteopontin) serves as a principal regulator of aberrant ECM remodeling and is prevalent in numerous conditions, including cancer, cardiovascular illness, pulmonary disease, chronic renal disease, and osteoarthritis. SPP1^+^ macrophages are pivotal biological agents of ECM dysregulation, facilitating collagen deposition, fibroblast activation, immunological modulation, and EMT. Targeting SPP1 and its interactions with ECM components presents a possible therapeutic approach to alleviate fibrosis, immunological evasion, and tissue dysfunction. Future research should concentrate on verifying these pathways *in vivo*, particularly in osteoarthritis, and enhancing the therapeutic use of SPP1-targeted medicines.

## Glossary

AKI-CKD: Acute Kidney Injury to Chronic Kidney Disease
Ang II: Angiotensin II
AR: Androgen Receptor
AS: Atherosclerosis
ASARM: Acidic, Serine- and Aspartic acid-Rich Motif
AAA: Abdominal Aortic Aneurysm
CAFs: Cancer-Associated Fibroblasts
CKD: Chronic Kidney Disease
COPD: Chronic Obstructive Pulmonary Disease
CRDs: Chronic Respiratory Diseases
CTLs: Cytotoxic T Lymphocytes
CVD: Cardiovascular Disease
DCM-HF: Dilated Cardiomyopathy with Heart Failure
DEHP: Di-(2-ethylhexyl) Phthalate
ECM: Extracellular Matrix
ecmCAFs: ECM-producing Cancer-Associated Fibroblasts
EMT: Epithelial-Mesenchymal Transition
GC: Gastric Cancer
GO: Gene Ontology
HCC: Hepatocellular Carcinoma
HDM: House Dust Mite
HNSCC: Head and Neck Squamous Cell Carcinoma
IFN-*γ*: Interferon-gamma
IL: Interleukin
ILT: Intraluminal Thrombus
IPF: Idiopathic Pulmonary Fibrosis
MAM: Matrisome-Associated Macrophage
MI: Myocardial Infarction
MMPs: Matrix Metalloproteinases
Mo-IMs: Monocyte-derived Interstitial Macrophages
mCRPC: metastatic Castration-Resistant Prostate Cancer
MSS-mCRC: Microsatellite Stable metastatic-type metastatic Colorectal Cancer
myCAFs: myofibroblastic Cancer-Associated Fibroblasts
NF-κB: Nuclear Factor Kappa-Light-Chain-Enhancer of Activated B Cells
NSCLC: Non-Small Cell Lung Cancer
OA: Osteoarthritis
PDAC: Pancreatic Ductal Adenocarcinoma
RPF: Rapid Pulmonary Fibrosis
RGD: Arg-Gly-Asp
scRNA-seq: Single-Cell RNA Sequencing
sEVs: Small Extracellular Vesicles
SIBLING: Small Integrin-Binding Ligand N-linked Glycoprotein
SNPs: Single-Nucleotide Polymorphisms
SPP1: Secreted Phosphoprotein 1
TAD: Thoracic Aortic Dissection
TAMs: Tumor-Associated Macrophages
TGF-*β*1: Transforming Growth Factor Beta 1
Th1: T-helper 1
Th17: T-helper 17 cells
TiME: Tumor Immune Microenvironment
TNBC: Triple-Negative Breast Cancer
TME: Tumor Microenvironment
VSMCs: Vascular Smooth Muscle Cells
